# An integrated newborn care kit (iNCK) to save newborn lives and improve health outcomes in Gilgit Baltistan (GB), Pakistan: study protocol for a cluster randomized controlled trial

**DOI:** 10.1186/s12889-023-17322-y

**Published:** 2023-12-11

**Authors:** Sarah M. Abu Fadaleh, Lisa G. Pell, Muhammad Yasin, Daniel S. Farrar, Sher Hafiz Khan, Zachary Tanner, Shariq Paracha, Falak Madhani, Diego G. Bassani, Imran Ahmed, Sajid B. Soofi, Monica Taljaard, Rachel F. Spitzer, Zulfiqar A. Bhutta, Shaun K. Morris

**Affiliations:** 1 Centre for Global Child Health, The Hospital for Sick Children, Toronto, ON Canada; 2 Gilgit Regional Office, Aga Khan Health Service – Pakistan, Gilgit-Baltistan, Pakistan; 3 Aga Khan Health Service – Pakistan, Karachi, Sindh Pakistan; 4 Brain and Mind Institute, Aga Khan University, Karachi, Sindh Pakistan; 5 Dalla Lana School of Public Health, University of Toronto, Toronto, ON Canada; 6 Department of Pediatrics, Temerty Faculty of Medicine, University of Toronto, Toronto, ON Canada; 7 Child Health Evaluative Sciences, The Hospital for Sick Children, Research Institute, Toronto, ON Canada; 8 Centre of Excellence in Women and Child Health, Aga Khan University, Karachi, Sindh Pakistan; 9 Clinical Epidemiology Program, Ottawa Hospital Research Institute, Ottawa, ON Canada; 10 School of Epidemiology and Public Health, University of Ottawa, Ottawa, ON Canada; 11 Department of Obstetrics and Gynaecology, University of Toronto, Toronto, ON Canada; 12 Section of Gynecology, The Hospital for Sick Children, Toronto, ON Canada; 13 Institute for Global Health & Development, The Aga Khan University, South-Central Asia & East Africa, Karachi, Pakistan; 14 Aga Khan University, Karachi, Sindh Pakistan; 15 Division of Infectious Diseases, The Hospital for Sick Children, 555 University Ave, Toronto, ON M5G 1X8 Canada

**Keywords:** Neonatal mortality, Gilgit-Baltistan, Pakistan, Newborn, Sepsis, Hypothermia, Omphalitis, Cluster randomized trial

## Abstract

**Background:**

Ongoing high neonatal mortality rates (NMRs) represent a global challenge. In 2021, of the 5 million deaths reported worldwide for children under five years of age, 47% were newborns. Pakistan has one of the five highest national NMRs in the world, with an estimated 39 neonatal deaths per 1,000 live births. Reducing newborn deaths requires sustainable, evidence-based, and cost-effective interventions that can be integrated within existing community healthcare infrastructure across regions with high NMR.

**Methods:**

This pragmatic, community-based, parallel-arm, open-label, cluster randomized controlled trial aims to estimate the effect of Lady Health Workers (LHWs) providing an integrated newborn care kit (iNCK) with educational instructions to pregnant women in their third trimester, compared to the local standard of care in Gilgit-Baltistan, Pakistan, on neonatal mortality and other newborn and maternal health outcomes. The iNCK contains a clean birth kit, 4% chlorhexidine topical gel, sunflower oil emollient, a ThermoSpot™ temperature monitoring sticker, a fleece blanket, a click-to-heat reusable warmer, three 200 μg misoprostol tablets, and a pictorial instruction guide and diary. LHWs are also provided with a handheld scale to weigh the newborn. The primary study outcome is neonatal mortality, defined as a newborn death in the first 28 days of life.

**Discussion:**

This study will generate policy-relevant knowledge on the effectiveness of integrating evidence-based maternal and newborn interventions and delivering them directly to pregnant women via existing community health infrastructure, for reducing neonatal mortality and morbidity, in a remote, mountainous area with a high NMR.

**Trial registration:**

NCT04798833, March 15, 2021.

**Supplementary Information:**

The online version contains supplementary material available at 10.1186/s12889-023-17322-y.

## Background

### The context of global and Pakistan specific neonatal mortality

Worldwide, more than 5 million under-five deaths were reported in 2021 of which 47% were newborns (i.e., those between 1 and 28 days of life, inclusive) [1]. Despite global efforts to reduce neonatal deaths, the reduction in mortality is not on track to meet the United Nations 2030 agenda for the Sustainable Development Goals (SDGs) global target of less than 12 neonatal deaths per 1,000 live births [1]. The main causes of neonatal mortality, many of which are preventable, include complications of preterm birth (36%), intrapartum-related complications (24%), infections (16%), and congenital abnormalities (10%) [2]. Further reduction in neonatal mortality requires that effective interventions are scaled up throughout the pre- and postnatal period [3], through innovative platforms and delivery strategies. The scale-up of peri- and postnatal interventions (e.g., skilled delivery attendants, neonatal hypothermia and hyperthermia monitoring, and umbilical cord care) has the potential to reduce newborn deaths related to infectious causes, conditions related to preterm birth, and intrapartum-related complications [3].

Pakistan has one of the highest neonatal mortality rates (NMR) globally. In 2021, reports estimated that 39 out of 1,000 live born newborns died within the first 28 days of life [1]. Pakistan's high NMR is in large part attributable to pervasive health challenges related to poverty and its associated barriers to accessing healthcare. For instance, with its diverse geography there are fewer health facility deliveries in Pakistan relative to other low- and middle-income countries (LMICs) and there is limited access to maternal and newborn healthcare services in rural and remote areas [4, 5]. In rural areas, 82% of pregnant women receive antenatal care (ANC) from healthcare professionals compared to 94% in urban settings [5]. Improving healthcare services in rural areas will require more robust ANC services, maternal education, monitoring programs for pregnancy-related adverse events, and facility deliveries attended by trained personnel [5]. The 2017–18 Pakistan Demographic and Health Surveys found that 41% of mothers in rural areas in Pakistan delivered at home. Furthermore, unsafe home delivery conditions and high-risk newborn practices were found to exacerbate newborn and maternal health risks [5]. These included non-sterile delivery and umbilical cord cutting practices, as well as the application of potentially harmful substances to the baby's skin and umbilical stump (e.g., ghee butter, ash etc.) [6, 7]. In addition to poor neonatal outcomes, Pakistan also has a high maternal mortality ratio (MMR) at 186 per 100,000 live births [8, 9], with the majority of maternal deaths related to postpartum hemorrhage (PPH) [8, 10].

### Healthcare infrastructure in Pakistan

In Pakistan, maternal and child healthcare services are provided through primary, secondary, and tertiary infrastructures that are both publicly and privately funded [11]. The public sector, including hospitals and health units, are well-appointed with trained staff and the necessary health supplies. However, due to factors such as long distances between homes and health facilities, limited working hours for healthcare workers, and financial limitations of both users and providers, the facilities are underutilized, particularly by those living in rural areas [8] which represents 64% of the total population [12]. In 1994, the government of Pakistan launched the Lady Health Worker (LHW) Program, a community health worker program that provides healthcare in rural communities to help address disparities in health services between rural and urban settings in Pakistan [13]. The LHW program aims to enhance access to healthcare services and improve health outcomes [5]. The program recruits LHW candidates from the local community who are between 18 and 45 years of age and have a minimum of eight years of education. LHWs receive training for 15 months in the prevention and treatment of common illnesses and how to deliver education about family planning and maternal and child health in their communities [5, 13]. LHWs are tasked with the following responsibilities: facilitate follow-up for ANC and immunization services, educate women about proper nutrition and disease treatments, promote safe hygienic practices, promote monitoring of children's health through regular home visits, provide family planning supplies, and provide essential preventive and curative health services (e.g., treatment and appropriate referral for children with diarrhea, fever, or respiratory infection) [13]. The LHW mandate is to conduct monthly household visits, including routine visits for pregnant women during their third trimester. Each LHW is responsible for about 150 homes (about 1000–1500 population) and is meant to visit on average 5–7 houses daily [13].

### Rationale

Interventions exist that have been shown to improve maternal and neonatal health and reduce mortality among at-risk populations [3]. However, these interventions are not always accessible or affordable, nor have they been packaged together to be delivered and used by caregivers at their homes. We hypothesize that complementary evidence-based interventions, when packaged together at low cost and delivered through existing community healthcare infrastructure, can lower neonatal and maternal death rates and reduce serious morbidities [3].

Between April 2014 and July 2015, we conducted a pilot community-based, cluster randomized, open-label, controlled intervention trial in Rahim Yar Khan (RYK), Punjab, Pakistan to estimate the effect of an integrated newborn care kit (iNCK) versus the local standard of care (control) on all-cause neonatal mortality [14]. The intervention group received the iNCK via LHWs that visited pregnant women at home during the third trimester of pregnancy. The iNCK included a clean birth kit (CBK) which contained a plastic sheet, cord clamps, sterile blade, gloves, and soap. The iNCK also included 4% chlorhexidine (CHX) topical gel, sunflower oil emollient, a continuous temperature monitoring device called ThermoSpot™, a Mylar reflective blanket, and a reusable instant warmer. We also provided battery-operated scales to LHWs in intervention clusters to measure the newborns' weight. The study enrolled 2663 pregnant women in the intervention group and 2788 pregnant women in the control group [14]. There was no statistically significant difference in NMRs between the intervention and control clusters (risk ratio [RR] 0.83, 95% confidence interval [CI] 0.58–1.18; *p* = 0.30). However, several key findings emerged from the study: (1) using existing community health infrastructure to distribute the iNCK was a feasible delivery mechanism for the intervention; (2) the risk of fever and omphalitis in the intervention clusters was lower compared to the control clusters (RR 0.64, 95% CI 0.47–0.87, *p* = 0.004; and RR 0.68, 95% CI 0.48–0.98, *p* = 0.04, respectively); (3) by using the portable battery-operated weighing scales, LHWs in the intervention group reliably identified a higher proportion of low-birthweight babies among all babies who were delivered at home, which prompted appropriate follow-up care for the newborns (RR 3.48, 95% CI 1.00–12.10; *p* = 0.05); (4) ThermoSpot™ enabled caregivers to identify and act on cases of fever, cold stress, and hypothermia; and, (5) while the iNCK was almost always used, caregiver compliance to specific instructions on how to use the iNCK was low (for example, only 32% and 28% of participants in intervention clusters who used CHX and ThermoSpot™, respectively, started use on the first day of the newborn’s life, as instructed) [14]. Based on the results generated in the RYK pilot trial, further investigation into the implementation of the iNCK is warranted. Key questions that remain unanswered include whether the provision of a pictorial instruction guide will improve caregiver compliance, whether the iNCK can be used to deliver important maternal health interventions, and if the iNCK could have a beneficial impact in a remote, low population density, high mortality setting that has a seasonally cold climate.

### Hypothesis

We hypothesize that using existing LHW networks to distribute the iNCK to pregnant women in Gilgit-Baltistan (GB), a region in northern Pakistan with high NMRs and limited access to facility-based healthcare, as well as enhanced education about correct use of the iNCK, will result in a significant reduction in newborn deaths compared to pregnant women who receive the local standard of care, but no iNCK.

## Methods

### Study setting

The study will be conducted in seven of the fourteen districts of Gilgit-Baltistan (GB): Astore, Diamer, Ghanche, Kharmang, Nagar, Shigar, and Skardu. Gilgit-Baltistan, located in northern Pakistan, bordered by China, Afghanistan, and India [15], has a terrain that is comprised of extensive mountain ranges and glaciers, making its geography and environment starkly different from the rest of Pakistan. Gilgit-Baltistan covers 72,971 km^2 ^and in 2017 had a population of 1.9 million people [15]. Most of the inhabitants of this region live in small, isolated villages that experience harsh weather conditions, particularly during the winter. These conditions further reduce accessibility to healthcare services in the area [15]. The 2017–18 Pakistan Demographic and Health Surveys estimated the NMR and under-five mortality rates in GB to be 47 deaths per 1,000 live births and 76 deaths per 1,000 live births [5], respectively. Additionally, only 64% of pregnant women gave birth with the help of a skilled provider, 78% had at least one problem accessing healthcare (e.g., management of transportation, distance to health facility, not wanting to go alone), and 38% gave birth at home [5].

### Study population

Pregnant women in their third trimester living within participating clusters and intending to be in the study area at the end of the neonatal period will be eligible for enrollment in this study.

### Study design

This study is a pragmatic, open-label, community-based, parallel-arm cluster randomized, controlled trial. Both the iNCK (intervention arm) and local standard of care (control arm) will be delivered to enrolled participants during their third trimester of pregnancy by LHWs. A total of 77 clusters, defined as union councils or sub-union councils with LHW coverage, were randomized, using covariate constrained allocation (using cluster specific estimates of NMR and proportion of health facility deliveries), to either the intervention or control arms. Union councils in Pakistan constitute district subdivisions directly involved in local government administration. These administrative divisions within the seven GB districts of interest were chosen as clusters because LHWs working within the territory of a certain union council provide services only to households within the boundaries of that union council. To obtain 77 clusters and reduce the risk of contamination, some larger union councils were further sub-divided into two or three sub-union councils, each of which was defined as a distinct study cluster based on LHW coverage (i.e., LHW(s) work only within the sub-union area and thus sub-unions could be randomized to different study arms). Union councils and sub-councils were treated as equal units when clusters were randomized to intervention or control groups. Finally, while 78 clusters were initially planned for randomization, one cluster in district Astore had to be excluded, before randomization, due to security concerns and therefore the final allocation was 39 clusters in the intervention arm versus 38 in the control arm.

The study will have two phases: 1) a baseline cross-sectional household survey; and 2) the interventional, cluster randomized trial. The baseline period was completed between June and August 2021, during which time survey data were collected from 10,264 households in the study catchment area. The data collected included information about birth history, stillbirths and neonatal deaths, PPH, maternal deaths, LHW coverage, migration patterns, phone ownership, and household experiences with COVID-19, including how the pandemic affected daily life. The survey was conducted using a two-stage, stratified sampling design and included participants from a representative sample of households from all 77 trial clusters. All data collected were used to ascertain cluster-specific statistics prior to the trial’s implementation such as birth rate, NMR, and the proportion of deliveries taking place at healthcare facilities across the region.

Following the baseline phase of the study and cluster randomization, field teams began to identify all new pregnancies throughout the study catchment area, and during the third trimester of pregnancy, approached these pregnant women to describe the study and collect written or identifying (fingerprint) informed consent for participation. Newly enrolled pregnant women in the intervention clusters are given iNCKs, and LHWs provide instructions on its proper use. Participant enrollment will continue for approximately 26 months to achieve target sample size. Data collectors are fully independent from LHWs and do not participate in any activities undertaken by LHWs in the field or facilities but are not blinded to cluster allocation as they ask questions about iNCK use from only participants in the intervention arm.

### Sample size

With the initially planned 39 clusters per trial arm, an average of 306 live births per cluster (23,868 pregnant women and live-born newborn pairs) is required to achieve 80% statistical power to detect a relative reduction of 25% in all-cause neonatal mortality. Sample size calculations were based on an assumed NMR of 35 newborn deaths per 1,000 live births in control clusters and an estimated intracluster correlation coefficient of 0.002 [16]. Furthermore, to allow for up to 5% attrition due to stillbirths and 10% attrition due to losses to follow-up, we aim to enroll a total of 27,448 pregnant women over a period of approximately 26 months.

### The iNCK Intervention and Standard of Care Comparator

**Fig. 1:**
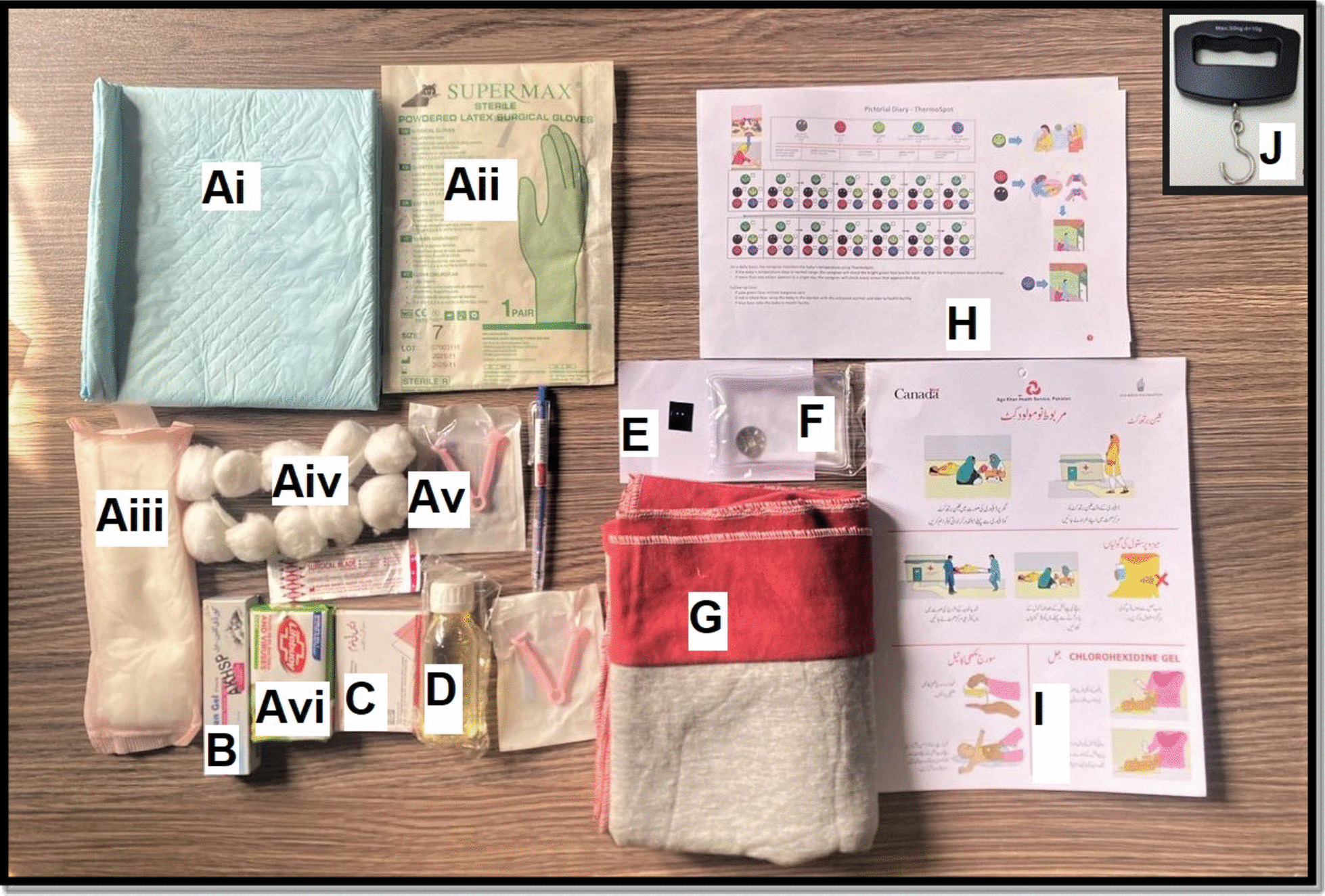
Contents of the iNCK, which includes: **A** Clean birth kit: Ai) Clean plastic sheet, Aii) surgical gloves, Aiii) maternity pad, Aiv) cotton balls, Av) cord clamp, Avi) antibacterial soap bar, **B** chlorhexidine 4% gel **C** three 200 μg misoprostol tablets, **D** sunflower oil emollient, **E** ThermoSpot™ sticker, **F** Click-to-heat warmer, **G** fleece blanket, **H** pictorial diary for documenting use of iNCK components, **I** pictorial guide, **J** battery-operated weighing scale (used by LHW)

The iNCK includes a clean birth kit, 4% CHX topical gel, sunflower oil emollient, one ThermoSpot™ temperature monitoring sticker, a fleece blanket, a reusable instant heat pack warmer, three 200 μg dissolvable sublingual misoprostol tablets, a pictorial instruction guide, and a pictorial diary to mark daily use of iNCK components (Fig. 1). All of these components are packaged into a large resealable transparent plastic bag that is delivered by LHWs to enrolled participants in the intervention group. Additionally, each LHW assigned to the intervention group was provided with one handheld battery-operated scale to weigh newborns at their first post-delivery home visit. Table 1 provides a detailed description of the individual iNCK contents and their intended use.

**Table 1:** Description of each iNCK component and its intended use

Kit content	Description and intended use
Clean birth kit (CBK)	**•**Sterile blade to cut the umbilical cord **•**Clean plastic sheet to be placed on the ground or **•**surface for birth **•**Plastic gloves to be worn by the birth attendant **•**Hand soap **•**Two cord clamps **•**Maternity pad to absorb peri- and post-natal bleeding **•**Ten cotton balls to apply CHX
Chlorhexidine (CHX)	**•**10 gm of 4% CHX topical gel, a broad-spectrum antiseptic, in a small plastic tube. CHX should be applied once per day from the first day of the baby’s life until day 10 of life
Misoprostol	**•**Three 200 µg dissolvable misoprostol tablets to be used prophylactically to prevent PPH. The tablets should be taken immediately after delivery of the baby and before delivery of the placenta. For participants who deliver via C-section, the attending clinician will decide whether or not to administer misoprostol
Sunflower oil emollient	**•**50 ml of sunflower oil emollient packaged in a small plastic bottle to massage over the newborn’s skin, avoiding the face and scalp. Sunflower oil emollient should be applied once per day from the first day of the baby's life until day 28 of life
ThermoSpot ™	**•**12 mm diameter round sticker that adheres to the skin and changes color based on body temperature. ThermoSpot™ should be applied to the baby’s neck over the carotid artery on the first day of the baby's life and left in place until day 14 of life
Fleece blanket	**•**One fleece blanket (65 × 86 cm), to wrap the baby along with the activated warmer whenever moderate to severe hypothermia is detected by ThermoSpot™
Warmer	**•**One reusable pocket-sized click-to-heat warmer that heats up to a temperature of about 50 °C and then gradually cools to ambient temperature over about 15 to 20 min. The warmer will be activated whenever moderate to severe hypothermia is detected by ThermoSpot ™
Pictorial guide	**•**A pictorial guide that illustrates how and when to use iNCK components
Pictorial diary	**•**The diary will enable self-reporting regarding compliance for individual iNCK components (i.e., If/when/how a component was used). Participants will tick boxes beside a series of images embedded in a calendar to indicate if she used a specific iNCK component on a given day. Each image contains a checkbox embedded in the upper right corner of the picture and corresponds to a day
Handheld battery-operated scale with a suspended cloth sling	**•**The scale will not be packaged within iNCK but will be distributed to LHWs in intervention clusters for measuring newborn weights, and prompting appropriate health facility visits for newborns found to be low birth weight (< 2500 g)

At the time of the third trimester delivery of the iNCK, the LHW will provide approximately 15–30 min of instructions to the pregnant women and other family members and caregivers who are present regarding the kit content and use. There were no further training sessions on how to use the iNCK delivered via the trial infrastructure; however, families had access to the pictorial iNCK instruction guide and could ask questions of LHWs during future household visits.

#### Clean birth kit (CBK)

Pregnant women will be instructed to provide the CBK to the birth attendant conducting the delivery, regardless of whether she delivers at home or at a facility. The CBK includes: i) Clean plastic sheet; ii) surgical gloves; iii) antibacterial bar of soap; iv) two cord clamps; v) maternity pad; and, and vi) cotton balls. A previous study in Pakistan reported a reduction in sepsis and neonatal mortality when combining CBK use with an educational component on how CBKs should be used [17]. A systematic review which included 37 studies (26 quantitative and 11 qualitative) from 15 countries (*N* = 48,767) found that the use of CBK was associated with a lowered occurrence of puerperal sepsis and a reduction in neonatal mortality [18].

#### Chlorhexidine (CHX)

The mother/caregiver will be taught to apply the CHX to a clean cotton ball and rub it on the newborn’s umbilical stump and the surrounding area, once per day, from the first day of the baby’s life until day 10 of life. In previous studies, the application of CHX to a baby’s umbilical cord showed a reduction in omphalitis and neonatal deaths in community settings [19, 20].

#### Misoprostol

The iNCK contains three 200 μg dissolvable misoprostol tablets. Mothers will be instructed to take the three tablets immediately after delivery of the baby and before delivery of the placenta to reduce the risk of PPH. For participants who deliver via C-section, the decision on misoprostol use will be deferred to the attending clinician. Data collectors will retrieve any unused misoprostol tablets from families at the home visit on day 29. Due to its low cost, oral route of administration, and no requirement for cold-chain storage, misoprostol can be used in low-income and remote settings and has been shown to be safe [21, 22].

#### Sunflower oil emollient

The mother/caregiver will be directed to massage a small amount of sunflower oil (Synergy Crafts, Karachi) onto the baby's skin all over their body, avoiding the face and scalp, once per day, from the day of birth until day 28 of life. In low-income countries, sunflower oil has been proven to be effective in preventing hospital-acquired infections in preterm neonates and supporting their critically compromised skin barrier [23].

#### Thermoindicator

The mother will be taught to place a ThermoSpot™ sticker (Maternova Inc, USA) over the carotid artery region of the newborn's neck on the day of birth and to use the sticker to continuously monitor the newborn’s body temperature until day 14 of life. She will be taught to reapply the sticker if it falls off before day 14. Additionally, she will be taught about the meaning of the different color responses, where a green face indicates normal temperature (36.5–37.5 °C), a pale green face indicates cold stress (36.0 °C–36.5 °C), a blue face indicates fever (> 39 °C), a red face indicates moderate hypothermia (35.0–35.5°C), and a black face indicates severe hypothermia (< 35°C). When the sticker turns pale green, the mother is educated to start kangaroo care, wherein she will place the baby skin-to-skin against her chest and monitor their temperature using the ThermoSpot™ sticker. Further, when the sticker turns red or black, the mother is instructed to place the newborn in the fleece blanket with the activated warmer and seek immediate medical care for the newborn. Caregivers will also be taught to seek immediate medical attention if the sticker turns blue, indicating that the baby has a fever. Several research studies have shown that ThermoSpot™ is effective in newborn temperature monitoring, especially in resource-limited areas [24, 25].

#### Fleece blanket and warmer

Whenever moderate to severe hypothermia is detected by ThermoSpot™, the mother is instructed to wrap the newborn with the provided, locally purchased, fleece blanket. The reusable instant heat pack warmer is to be activated when the ThermoSpot™ detects that the newborn is experiencing moderate or severe hypothermia. Inside the warmer is a small metal disk that, when flexed, begins a rapid exothermic crystallization reaction of the surrounding salt solution. Once activated, the contents of the warmer will begin to solidify and will increase in temperature to about 50 °C and then gradually cool to ambient temperature over a period of about 15–20 min. The solidified solution can later be returned to a liquid form by boiling the warmer in water.

#### Pictorial guide

A pictorial guide was developed locally to be culturally appropriate and contains non-written, step-by-step illustrations showing how to use the iNCK components. The pictorial guide is inserted into the resealable bag to ensure it remains with the iNCK and is not lost. The purpose of the pictorial guide is to improve compliance with the intended use of each iNCK component in a setting with low literacy rates.

#### Pictorial diary

The mother/caregiver will be provided with a pictorial diary to self-report their use of individual iNCK components using a simple check-box approach that does not require users to be able to write. LHWs will provide instructions on how to use the pictorial diary during the third trimester visit. The diaries will be collected from participants during the 29th-day visit.

#### Handheld battery-operated scale with a suspended cloth sling

LHWs will weigh the newborn during their first post-delivery home visit. The neonate will be weighed twice, and the average of these two weights will be recorded as the birth weight. The day on which the LWH measures the newborn’s weight will be recorded.

### Randomization

Covariate-constrained randomization was used to promote balance in key indicators associated with neonatal mortality between arms [26]. The randomization strategy considered: (1) cluster-level baseline NMR; (2) cluster-level baseline proportion of newborns delivered at a health facility; and, (3) GB study district (nominal categorical variable of the seven study districts). In December 2021, all clusters were randomized and allocated to either the intervention or control arm by a statistician who is not affiliated with the study, using the cvcrand package in R version 4.1.2 [27]. Balanced randomization schemes were defined as those where between-arm differences for baseline NMR were < 5%, baseline health facility deliveries were < 10%, and where at least 33% of clusters per district were allocated to each arm. The statistician then randomly selected one scheme from all balanced schemes (*n* = 11,041) using a random seed number.

### Delivery of the integrated neonatal care kit

As part of their routine activities, LHWs identify pregnant women and record the first day of their last menstrual period. As part of their mandate, LHWs already register all pregnant women in their catchment area within the first trimester of pregnancy. On a monthly basis, LHWs report the number of newly identified pregnancies to their Lady Health Supervisor (LHS).

To efficiently capture pregnancy data and enable timely iNCK delivery and subsequent data collection, our research team will provide LHSs across each of the seven districts with a pregnancy identification form to document the pregnant woman’s name, address, and phone number. LHSs will distribute these forms to LHWs, who will in turn complete them during home visits when they first identify new pregnancies. The information enables study-employed data collectors to locate the appropriate households to screen for eligibility, obtain written consent, and enroll participants, and for field supervisors to contact the mothers/caregivers directly to schedule data collection visits.

During this same visit, and after collecting written informed consent, LHWs will provide the iNCKs to participants in intervention clusters, educate them on how to use the kit components plus provide the local standard of care. The LHW will be directly observed during these activities by a trained data collector who will complete an evaluation of the process. Participants who reside in control clusters will not receive iNCKs but will receive the local standard of care from LHWs. To facilitate the timely and reliable notifications of new births, at the time of consent, all participants will be provided with contact information and instructions to notify the study team and/or their LHWs as soon as possible following delivery.

### Study outcome measures

The primary outcome measure is all-cause neonatal mortality, defined as the death of a live-born child from any cause within the first 28 days of life. Study outcomes (Table 2) will be measured on all enrolled participants.

**Table 2:** Primary and secondary outcomes

Primary Outcome	**•**All-cause neonatal mortality among live born neonates in first 28 days of life
Secondary Outcomes	**•**Cumulative incidence of omphalitis
	**•**Cumulative incidence of post-partum hemorrhage (PPH)
	**•**Post-delivery health facility utilization by pregnant women, and/or neonates and mothers in the first month postpartum
	**•**Cumulative incidence of hypothermia within the first 28 days of life, as identified through use of the ThermoSpot™, and explore the seasonal variability (by month) in the cumulative incidence of hypothermia **•**Cumulative incidence of fever within the first 28 days of life, as identified through use of the ThermoSpot™
	**•**Compliance with instructions on how to use the iNCK
	**•**Cause-specific neonatal mortality as determined by verbal autopsy

### Data collection and data management

Data collectors who have a minimum qualification of a high school diploma will work independently from the LHWs to collect baseline and trial data. They will undergo a three day instructional training session covering the trial’s design, the iNCK components, and all data collection forms.

During the implementation of the baseline survey, data collectors visited selected villages from the included clusters, explained the purpose of the study to the sampled households, and administered baseline questionnaires, the results of which were used to calculate the region and cluster-specific NMRs and birth rates.

For the intervention phase of the trial, data collectors will complete one in-person at-home visit, a mid-point assessment call, and try for a second in-person at home visit. The first in-person visit will occur during the third trimester of pregnancy, at which time data collectors will screen potential participants for eligibility. If eligibility criteria are met, participants who provide informed consent to participate in the trial will receive a pictorial diary from the LHW to enable self-reporting regarding compliance for individual iNCK components (intervention clusters only). Data collectors will then administer a questionnaire in the form of a structured interview to collect sociodemographic and household data. On the 8^th^ day of a newborn's life, field supervisors will attempt to complete phone interviews with participant households to collect information surrounding the baby's delivery and study outcomes of interest that may have occurred during the first week of the newborn’s life. While efforts will be made to complete these calls on the 8th day of life, a one-week window will be permitted for completing this phone call (between the 8th day through the 14th day of the newborn’s life). If the study team is unable to contact the participant for this data collection touchpoint, data collectors will obtain this information during the in-person at-home visit on the 29th day of the baby’s life. Data collectors will visit participant households on day 29 of the newborn's life to collect information that will enable the assessment of primary and secondary outcomes related to neonatal and maternal health, and will collect the completed pictorial diaries and any unused misoprostol tablets from the participants before leaving. While efforts will be made to collect data on day 29, this in-person visit will be attempted up until day 43 post-natal age, if necessary. If, after two to three consecutive visits, the study team cannot reach a participant household by day 43, the day 29 questionnaire may be completed over the phone by the field supervisor that presides over the districts in which these participants live. However, if a participant cannot accommodate the time required to complete the questionnaires involved with the day 29 visit by phone or in person before or on day 43 of the newborn’s life, or if the participant is lost to follow-up following the notification of birth, a modified vital outcomes questionnaire will be administered to participants, if possible, either in-person or over the telephone, to confirm whether their baby survived the neonatal period.

In the event of a newborn death or stillbirth, data collectors will request permission to administer a verbal autopsy (VA) to ascertain the cause of death or stillbirth. The World Health Organization’s guidelines on the development and use of VA are being used in this study with adaptations for the local context [28]. A minimum 2-week mourning period will precede administration of the VA.

To manage and store data collection forms throughout the trial’s duration, a main hub office in Gilgit and a sub-hub office Skardu as well as and six "spoke" offices in Ghanche, Kharmang, Shigar, Nagar, Astore, and Diamer have been established to coordinate the collection of data and the transfer of completed data collection forms. All data will be collected on paper forms. Data collectors administer the questionnaires, collect completed forms and diaries from participants, and deliver these items back to the Skardu or Gilgit offices within one week of collecting data. Forms and diaries are transferred to the Skardu office from districts Ghanche, Kharmang, Shigar, and Skardu, while completed forms are transferred to the Giglit office from districts Nagar, Astore, and Diamer. To ensure data security, a supervisor or data collector will travel with the paper forms in a study vehicle and conduct an in-person handover process with the teams in Skardu and Gilgit. Upon receiving completed forms in each district office, the field supervisor will review them, and if a potential error is identified, will contact the data collector who interviewed the participant to assess and, if needed, reconcile the error and sign off on the accuracy of the form. Data entry specialists will assess the paper forms for completeness and consistency. If any issues are discovered, these will be discussed with the project team and respective field supervisors. The field supervisor will consult the original data collectors for possible explanations. All forms will be double-entered into a REDCap database and compared for errors. Discrepancies between the two entries will be reconciled by referring to the original paper forms.

### Statistical analysis plan

Hypothesis testing for this study will be based on a superiority framework and will be conducted at the α = 0.05 significance level. Primary analyses will be conducted using a complete-case, intention-to-treat approach, where participants will be analyzed in the trial arm their cluster was randomized to, irrespective of contamination or adherence to instructions on how to use the iNCK. The unit of analysis will be the individual neonate (neonatal mortality, omphalitis, fever, hypothermia, and healthcare utilization for newborn morbidities) or the individual woman (PPH and healthcare utilization for PPH). Risk ratios for the primary (neonatal mortality) and secondary (omphalitis and PPH) outcomes will be estimated using multivariable modified Poisson regression [29], with generalized estimating equations to account for the clustered design and robust variance estimators to account for multiple births and/or multiple pregnancies during the study period. Analyses of neonatal mortality (the primary outcome) will adjust for newborn sex, antenatal care, and gestational age at birth to improve power and efficiency; calendar time to account for underlying temporal trends (defined as month of birth, setting month of study launch as month 0); and the covariates used in the randomization procedure: GB study district, log cluster-specific baseline NMRs, and the cluster-specific baseline proportion of children delivered at a health facility. Analyses of omphalitis (secondary outcome) will adjust for newborn sex, antenatal care, and GB study district. Analyses of PPH (secondary outcome) will exclude women who delivered via C-section, and will adjust for parity, maternal age, history of prior C-section, history of prior PPH, GB study district, and the cluster-specific baseline proportion of women who reported experiencing PPH. The frequency of hypothermia, cold stress, and fever detected by ThermoSpot™ within the first 14 days of life will be described amongst participants in intervention clusters only, overall and by calendar month to assess seasonality. Finally, the frequency and location of health facility utilization for omphalitis, PPH, hypothermia, and fever will be described using counts and proportions amongst individuals experiencing each respective morbidity and will be stratified by trial arm. The proportion accessing healthcare services by trial arm will be compared using clustered t-tests and modified Poisson regression.

Subgroup analyses will examine primary and secondary outcomes by place of delivery, GB district, newborn sex, gestational age at birth, the timing of intervention delivery relative to the date of birth, and the frequency of postnatal LHW home visits (Table 2). For analyses of neonatal mortality, omphalitis, and PPH, interaction terms between trial arm and each subgroup or process indicator will be analyzed in separate models. We will also describe compliance to the iNCK package and its components among intervention arm participants, including overall uptake as well as the timing and duration of usage. In secondary, per-protocol analyses of the primary and secondary outcomes, intervention arm participants will be assigned compliance scores using an algorithm defined in Supplemental Tables 1 and 2. Participants scoring at or above thresholds of compliance (e.g., 60%, 70%, 80%, 90%, and 100%) will be propensity-score matched to participants in control clusters, and modified Poisson regression will be used as above to compare good compliers with their matched unexposed participants. Finally, we will conduct supplementary analyses of the primary neonatal mortality outcome using alternative mortality definitions (i.e., death on day 1, days 1–7, days 8–28, days 2–7, and days 2–28).

Cause of death will be ascertained using VA data using previously described methods and will also inform the primary analysis by differentiating stillbirths from early neonatal deaths [30]. Stata version 17.0 or newer will be used for all analyses.

### Data monitoring & interim analysis

A data and safety monitoring board (DSMB) comprised of professionals with expertise in neonatal and child health will oversee the safety of the trial. Each DSMB member will be independently responsible for making recommendations for stopping the study based on specific safety endpoints.

Biannual interim analyses will examine the primary and secondary outcomes as well as iNCK compliance measures. The DSMB will convene annually, and at least two weeks prior to each meeting, the panel members will be given a written report outlining the current status of the study, masked outcome data, and information pertaining to any adverse events that may have occurred. If the mortality difference becomes higher than 40% in either direction (i.e., 40% greater mortality in the intervention or control clusters), the DSMB will be unmasked, and a decision will be made on whether the trial should continue.

### Adverse event reporting

The study will employ a passive adverse event reporting system. Should any potential adverse event be reported to the study team, a data collector or field supervisor will complete an Adverse Event Report Form and document the participant-reported details surrounding the event. This form will be electronically sent by the field supervisor to a study physician at Aga Khan Health Services, Pakistan (AKHS,P) for evaluation. If deemed to be study-related, the study physician will inform the principal investigator. Adverse events will be reported to the required regulatory bodies within 15 calendar days from the day the principal investigator becomes aware. The principal investigator will notify the DSMB about study related adverse events and, if deemed necessary, the DSMB will make recommendations to the principal investigator about stopping the trial.

### Results dissemination

Throughout the project, regular communication with local government and other regional stakeholders will take place to share progress updates. Interim and final trial results will also be communicated to AKHS,P and GB district health leadership. The study team will recommend that GB district health leadership share findings from the study with LHSs, who will relay results to LHWs, who may then communicate findings to households across GB during home visits.

### Confidentiality

Information obtained in the study will be securely transmitted, and the identity of the subjects will remain confidential. All records will be kept confidential in keeping with requirements as stipulated by the ethics boards of Aga Khan University, the National Bioethics Committee of Pakistan, and the Hospital for Sick Children.

## Discussion

Despite global efforts to reduce neonatal mortality, reductions in NMR remain behind what is needed to reach the SDG global target of less than 12 neonatal deaths per 1,000 in 2030 [1]. Interventions that target the most common causes of newborn deaths in high burden countries, and that are available from the time of delivery, including for home-delivered births, are crucial to reaching SDG targets [3].

GB, the northernmost administrative territory in Pakistan, has high NMRs and a large proportion of its population live in rural and remote areas. This study will deliver a portable, low-cost, easy-to-use, and evidence-based iNCK to pregnant women in GB and empower them with the knowledge needed to use the iNCK. This innovative intervention has the potential to meaningfully reduce mortality and morbidity in newborns. Moreover, utilizing Pakistan’s existing community health infrastructure to deliver the iNCK to pregnant women will inform its scale-up in similar settings with high NMR and community health worker programs.

### Supplementary Information

**Additional file 1.**

** Additional file 2.**

## Data Availability

Upon completion of the study, the datasets used and/or analysed during the current study are available from the corresponding author on reasonable request and with a data sharing agreement.
