# Supplementary material for: An integrated newborn care kit (iNCK) to save newborn lives and improve health outcomes in Gilgit Baltistan (GB), Pakistan: study protocol for a cluster randomized controlled trial
Source: BMC Public Health. 2023 Dec 11;23:2480. doi: 10.1186/s12889-023-17322-y (PMC10714624; doi:10.1186/s12889-023-17322-y)
Supplement: Supplementary file 1 — Additional file 1. [file 12889_2023_17322_MOESM1_ESM.docx]

**Supplemental Table 1.** Compliance scores for newborn interventions, defined by individual iNCK component.

|  | | 0 | 1 | 2 | 3 |
| --- | --- | --- | --- | --- | --- |
| **iNCK Component** | | **Never Used** | **Partial Compliance** | **Good Compliance** | **Excellent Compliance** |
| **Clean Birth Kit (CBK)** | | Did not use the CBK **OR** Did not know if the CBK was used **AND** Delivery happened at home | Did not use the CBK **OR** Did not know if the CBK was used **AND** Delivery happened at "other" (i.e., traditional birth attendant’s house, house) | Did not use the CBK **OR** Did not know if the CBK was used **AND** Delivery happened at primary health care facility (i.e., BHU, RHC, private clinic, or Dispensary) | Used the CBK at the time of delivery; **OR**  Did not use the CBK **OR** Did not know if the CBK was used **AND** delivery happened at hospital (THQ, DHQ) |
| **Chlorhexidine (CHX)** | | **Newborns that Survived Neonatal Period**:  Never applied CHX to umbilical stump  **Newborns that did NOT Survive Neonatal Period:**  Never applied CHX to umbilical stump **AND**  Newborn did not die on day 1 | First applied CHX to umbilical stump after day 1 (regardless of neonatal outcome of baby) | **Newborns that Survived Neonatal Period:**  Applied CHX to the umbilical stump on day 1 and applied it for less than 10 days **OR** stopped application before the cord separated (if the cord separated before day 10)  **Newborns that did NOT Survive Neonatal Period:** Applied CHX to the umbilical stump on day 1 and applied it for less than 10 days **OR** they stopped before the day the cord separated (if the cord separated before day 10) **AND** they stopped applying CHX before the day the baby died | **Newborns that Survived Neonatal Period:** Applied CHX to the umbilical stump on day 1 and applied it for at least 10 days **OR** greater than or equal to the day that the cord separated   **Newborns that did NOT Survive Neonatal Period:** Applied CHX to the umbilical stump on day 1 and applied it for at least 10 days **OR** greater than or equal to the day the cord separated **OR** until the day that the baby died; **OR** never applied CHX and baby died on day 1 |
| **Sunflower Oil** | | **Newborns that Survived Neonatal Period**: Did not apply sunflower oil  **Newborns that did NOT Survive Neonatal Period:** Did not apply sunflower and baby died after day 1 | **Newborns that Survived Neonatal Period:** Applied sunflower oil but stopped before day 7 (regardless of start date)  **Newborns that did NOT Survive Neonatal Period:** Applied sunflower oil but stopped before day 7 (regardless of start date) and stopped using oil before the day that the baby died | **Newborns that Survived Neonatal Period:** First applied oil on day 1 and stopped applying it on at least day 7  **Newborns that did NOT Survive Neonatal Period:** First applied oil on day 1 and stopped applying oil on at least day 7 (but before day 14) and before the day that the baby died | **Newborns that Survived Neonatal Period:** First applied sunflower oil on day 1 **AND** stopped applying oil on day 7 or later   **Newborns that did NOT Survive Neonatal Period:** First applied sunflower oil on day 1 **AND** applied oil until at least day 7 **OR** until the day baby died (if they died before day 7) **OR** Did not apply oil and baby died on day 1 |
|  | | **0** | **1** | **2** | **3** |
| **iNCK Component** | | **Never Used** | **Partial Compliance** | **Good Compliance** | **Excellent Compliance** |
| **ThermoSpot (TS)**  **Utilization** | | **Newborns that Survived Neonatal Period**: Did not use TS  **Newborns that did NOT Survive Neonatal Period**: Did not use TS and baby did not die on day 1 | **Newborns that Survived Neonatal Period:** First applied TS *after day 3* (irrespective of when they stopped); **OR** First applied TS on day 1 and stopped using it before day 4 (i.e., only 3 days of usage); **OR** First applied TS on day 2 or day 3 and stopped using it before day 6 or 7, respectively (i.e., only 4 days of usage)  **Newborns that did NOT Survive Neonatal Period**: First applied TS on day 1 and stopped using it before day 4 (i.e., only 3 days of usage) and baby died after they stopped using TS; **OR** First applied TS on day 2 or day 3 and stopped using it before day 6 or 7, respectively (i.e., only 4 days of usage) and baby died after they stopped using TS | **Newborns that Survived Neonatal Period**: First applied TS on day 1 and stopped using it on or after day 4 (i.e., at least 4 days of usage that started on day 1); **OR** First applied TS on day 2 or day 3 and stopped using it on or after day 6 or day 7, respectively (i.e., at least 5 days of usage)  **Newborns that did NOT Survive Neonatal Period**: First applied TS on day 1 and stopped using it on or after day 4 (i.e., at least 4 days of usage that started on day 1) and they died after they stopped using TS; **OR** First applied TS on day 2 or day 3 and stopped using it on or after day 6 or day 7, respectively (i.e., at least 5 days of usage) and they died after they stopped using TS; **OR** First applied TS on day 2 or day 3 and stopped using TS on the day baby died | **Newborns that Survived Neonatal Period**: First applied TS on day 1 and used it until at least day 7  **Newborns that did NOT Survive Neonatal Period**: First applied TS on day 1 and used it until at least day 7 or until the day they died; **OR** Did not use TS and baby died on day 1 |
|  | **Red:**  **Moderate Hypothermia** | None of the following:  Used warmer  Used blanket  Sought health care | Any one of the following:  Used warmer  Used blanket  Sought health care | Any two of the following:  Used warmer  Used blanket  Sought health care | All three of the following:  Used warmer  Used blanket  Sought health care |
| **Thermal**  **Action^1^** | **Black:**  **Severe Hypothermia** | None of the following:  Used warmer  Used blanket  Sought health care | Any one of the following:  Used warmer  Used blanket  Sought health care | Any two of the following:  Used warmer  Used blanket  Sought health care | All three of the following:  Used warmer  Used blanket  Sought health care |
|  | **Compliance Score** | **0 - Never Used** | | **3- Excellent Compliance** | |
|  | **Pale Green: Cold Stress** | Did not exercise Kangaroo care | | Exercised Kangaroo care | |
|  | **Blue: Fever** | Did not seek health care | | Sought health care | |

^1^Participants who do not experience cold stress, hypothermia, or fever (i.e., and not requiring usage of these components) will be assigned a score of 3, indicating perfect compliance to the Thermal Action components.

**Supplemental Table 2.** Compliance scores for misoprostol.

| 0 | 0 | 1 | 2 |
| --- | --- | --- | --- |
| **Never Used** | **Used Before Baby’s Delivery** | **Partial Compliance** | **Excellent Compliance** |
| Did not use any misoprostol  OR  For facility births: Did not bring the misoprostol tablets to the facility where the birth took place | Administered any quantity of tablets before delivery of the baby  OR  For twin/multiple births: Administered any quantity of misoprostol before the delivery of the second/final baby | Administered one or two tablets between delivery of the baby and delivery of the placenta AND did not use the remaining tablet(s) OR administered the remaining tablet(s) following delivery of the placenta  OR  Administered all three tablets following delivery of the placenta | Administered all three tablets between delivery of the baby and delivery of the placenta  OR  For facility births: Brought the misoprostol tablets to the facility where the birth took place |
