# Supplementary material for: An integrated newborn care kit (iNCK) to save newborn lives and improve health outcomes in Gilgit Baltistan (GB), Pakistan: study protocol for a cluster randomized controlled trial
Source: BMC Public Health. 2023 Dec 11;23:2480. doi: 10.1186/s12889-023-17322-y (PMC10714624; doi:10.1186/s12889-023-17322-y)
Supplement: Supplementary file 2 — Additional file 2. [file 12889_2023_17322_MOESM2_ESM.docx]

**Supplemental Figure.** Data Collection: Overview of Forms Completed at Each Participant Touchpoint

**During pregnancy**:


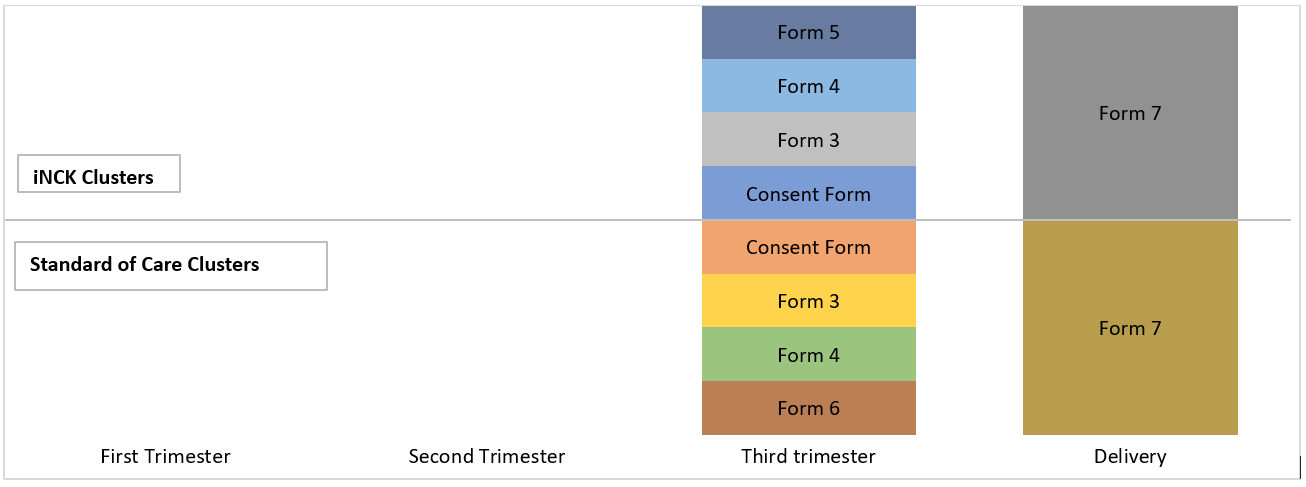


| **Form Number** | **Form Name** |
| --- | --- |
| Form 3 | Participant eligibility screening form |
| Form 4 | Demographic and household data |
| Form 5 | LHW iNCK teaching checklist |
| Form 6 | LHW local standard of care teaching checklist |
| Form 7 | Birth notification form |

**Post-natal Days:**


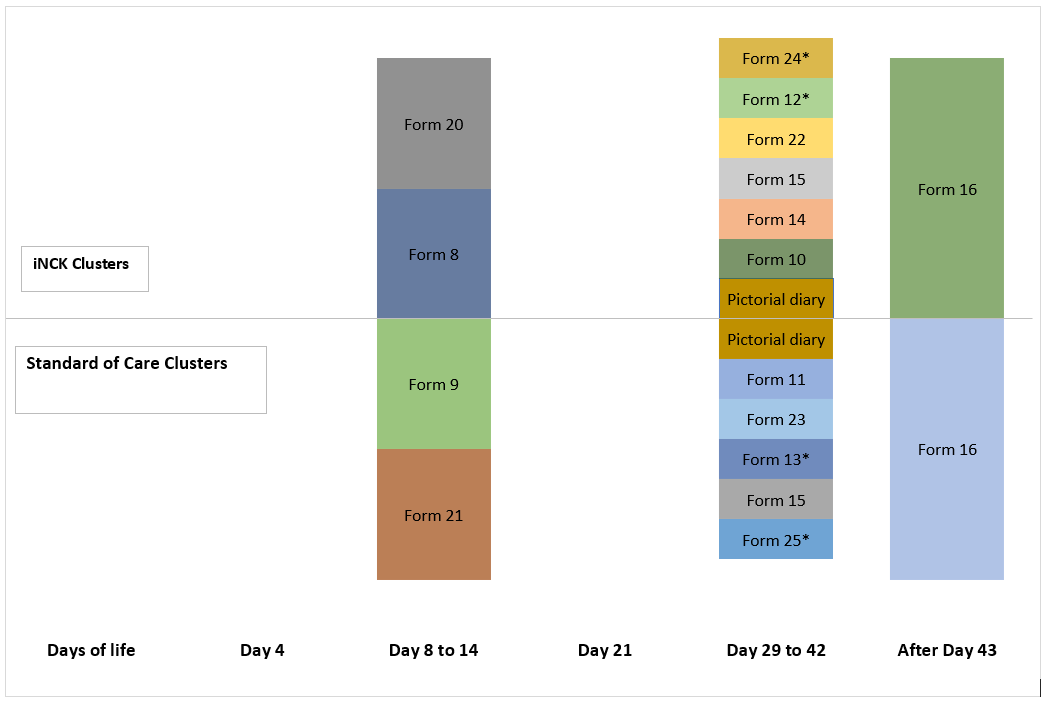


| **Form Number** | **Form Name** |
| --- | --- |
| Form 8 | Day 8 phone call questionnaire |
| Form 9 | Day 8 phone call questionnaire |
| Form 20 | Day 8 phone call questionnaire for multiple births |
| Form 21 | Day 8 phone call questionnaire for multiple births |
| Form 10 | Day 29 outcomes questionnaire |
| Form 11 | Day 29 outcomes questionnaire |
| Form 12 | Day 29 outcomes questionnaire (* if the day 8 call was not completed) |
| Form 13 | Day 29 outcomes questionnaire (* if the day 8 call was not completed) |
| Form 22 | Day 29 outcomes questionnaire for multiple births |
| Form 23 | Day 29 outcomes questionnaire for multiple births |
| Form 24 | Day 29 outcomes questionnaire for multiple births (* if the day 8 call was not completed) |
| Form 25 | Day 29 outcomes questionnaire for multiple births (* if the day 8 call was not completed) |
| Form 14 | Acceptance and willingness to pay for iNCK |
| Form 15 | Verbal Autopsy form |
| Form 16 | Vital outcomes questionnaire |
